# Supplementary material for: The careg element reveals a common regulation of regeneration in the zebrafish myocardium and fin
Source: Nat Commun. 2017 May 3;8:15151. doi: 10.1038/ncomms15151 (PMC5418624; doi:10.1038/ncomms15151)
Supplement: Supplementary Information — Supplementary Figures. [file ncomms15151-s1.pdf]

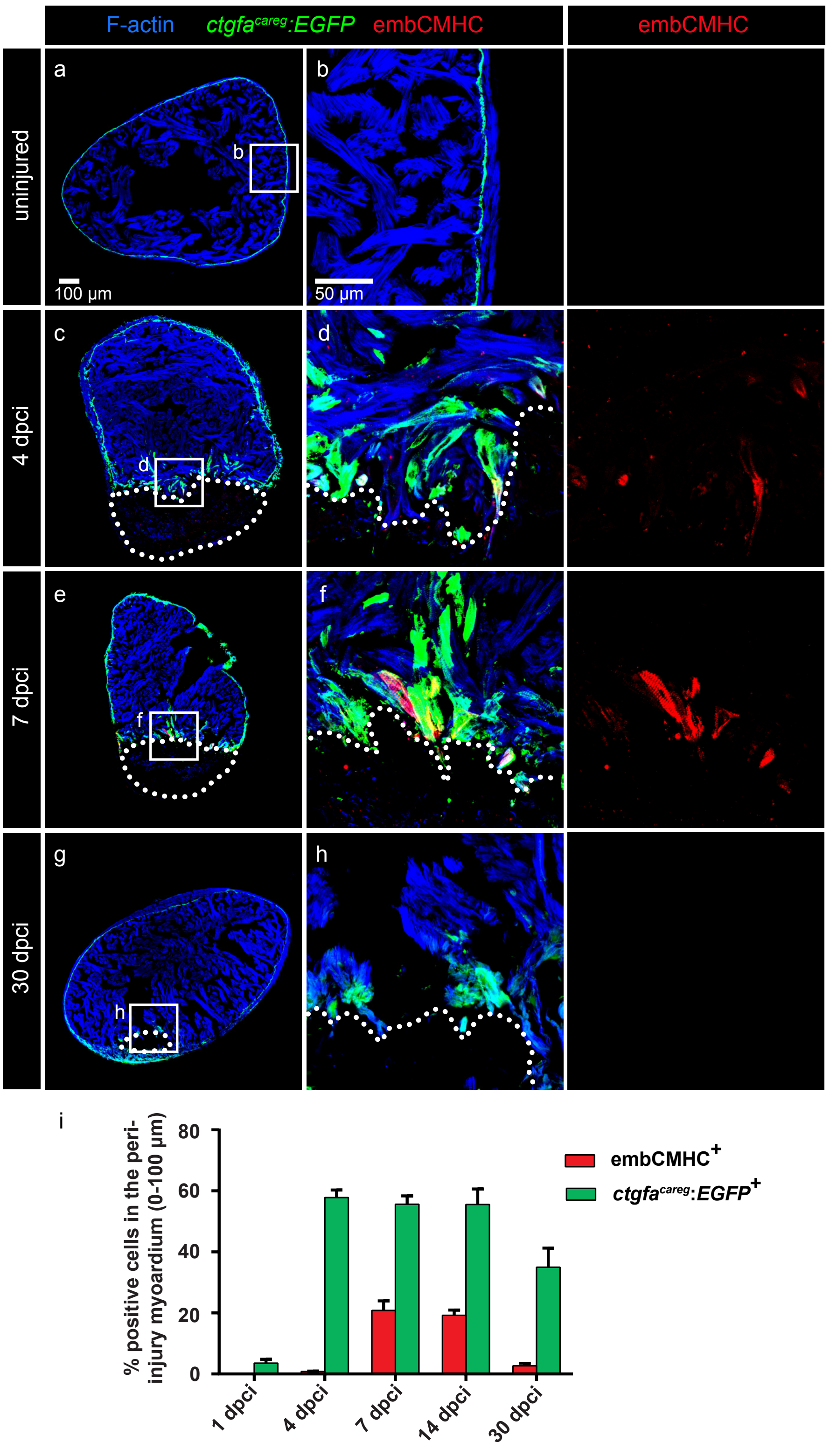

F-actin ctgfa careg:EGFP embCMHC
embCMHC
uninjured
4 dpci
7 dpci
30 dpci
a
b
c
d
e
f
g
h
100 µm
50 µm
i
% positive cells in the peri-injury myoardium (0-100 µm)
0
20
40
60
80
1 dpci
4 dpci
7 dpci
14 dpci
30 dpci
embCMHC+
ctgfa careg:EGFP+

Supplementary Figure 1. The *ctgfa^{careg}:EGFP* transgenic reporter is transiently activated in the peri-injured myocardium during heart regeneration.

(a-h) Immunofluorescence staining of *ctgfa^{careg}:EGFP* transversal heart sections with antibodies against GFP (green) and embCMHC (red) at different time points after cryoinjury. The intact myocardium is detected by F-actin staining (Phalloidin, blue). (a-b) In uninjured ventricle, *ctgfa^{careg}:EGFP* is expressed in a fine layer in the subcortical myocardium. No embCMHC is observed. (c-d) At 4 dpci, *ctgfa^{careg}:EGFP* is induced in trabecular fascicles abutting the post-infarcted area, a subset of which also displays embCMHC expression. (e-f) At 7 dpci, *ctgfa^{careg}:EGFP* expression is maintained at the injury border covering embCMHC+ CMs. (g-h) At 30 dpci, *ctgfa^{careg}:EGFP* is reduced to a small margin around the remaining fibrotic tissue. (i) Quantification of *ctgfa^{careg}:EGFP*+ and embCMHC+ area within 100 µm from the injury border. N ≥ 8.

Post-infarcted ventricle is encircled with a dotted line. The same rules apply to all subsequent figures.

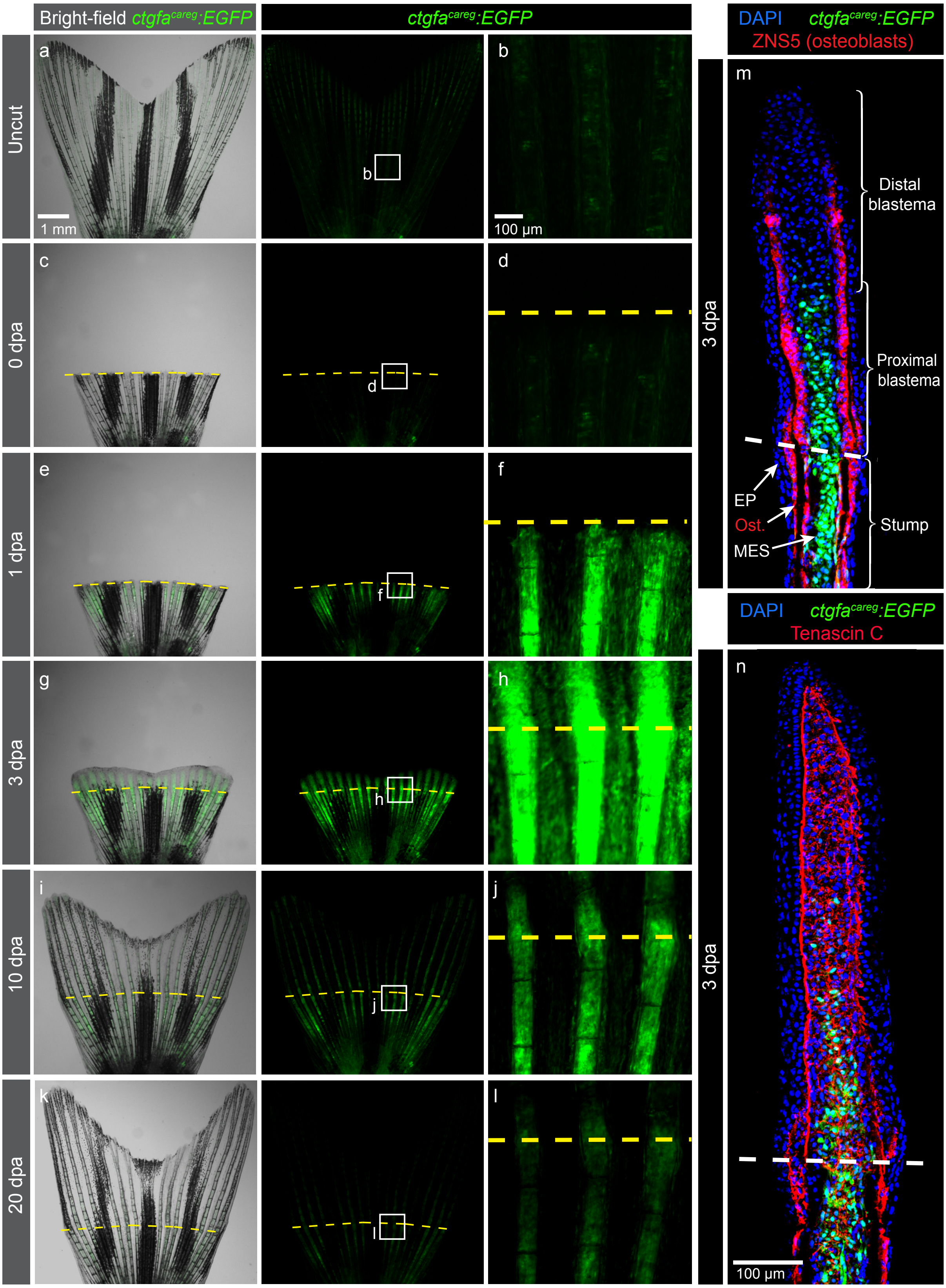
Bright-field ctgfa careg:EGFP
ctgfa careg:EGFP
Uncut
0 dpa
1 dpa
3 dpa
10 dpa
20 dpa
a
b
c
d
e
f
g
h
i
j
k
l
1 mm
100 μm
DAPI ctgfa careg:EGFP
ZNS5 (osteoblasts)
m
3 dpa
Distal blastema
Proximal blastema
Stump
EP
Ost.
MES
DAPI ctgfa careg:EGFP
Tenascin C
n
3 dpa
100 μm

Supplementary Figure 2. Peri-injury tissue of the fin stump transiently induces *ctgfa^{careg}:EGFP* expression

(a-l) Live imaging of *ctgfa^{careg}:EGFP* fins at different days post-amputation (dpa). Higher magnifications of framed areas show the region of the amputation plane (yellow dashed line). N=4.

(m-n) Immunofluorescence staining of longitudinal *ctgfa^{careg}:EGFP* fin sections at 3 dpa. EP, epidermis; Ost, osteoblasts; MES, mesenchmyme. (m) *ctgfa^{careg}:EGFP* is detected in the stump mesenchyme and osteoblasts (red) below the amputation plane (dashed line), and in the proximal blastema. (n) The mesenchyme of the regenerating fin abundantly expresses Tenascin C, a tissue remodelling extracellular protein. N≥4.

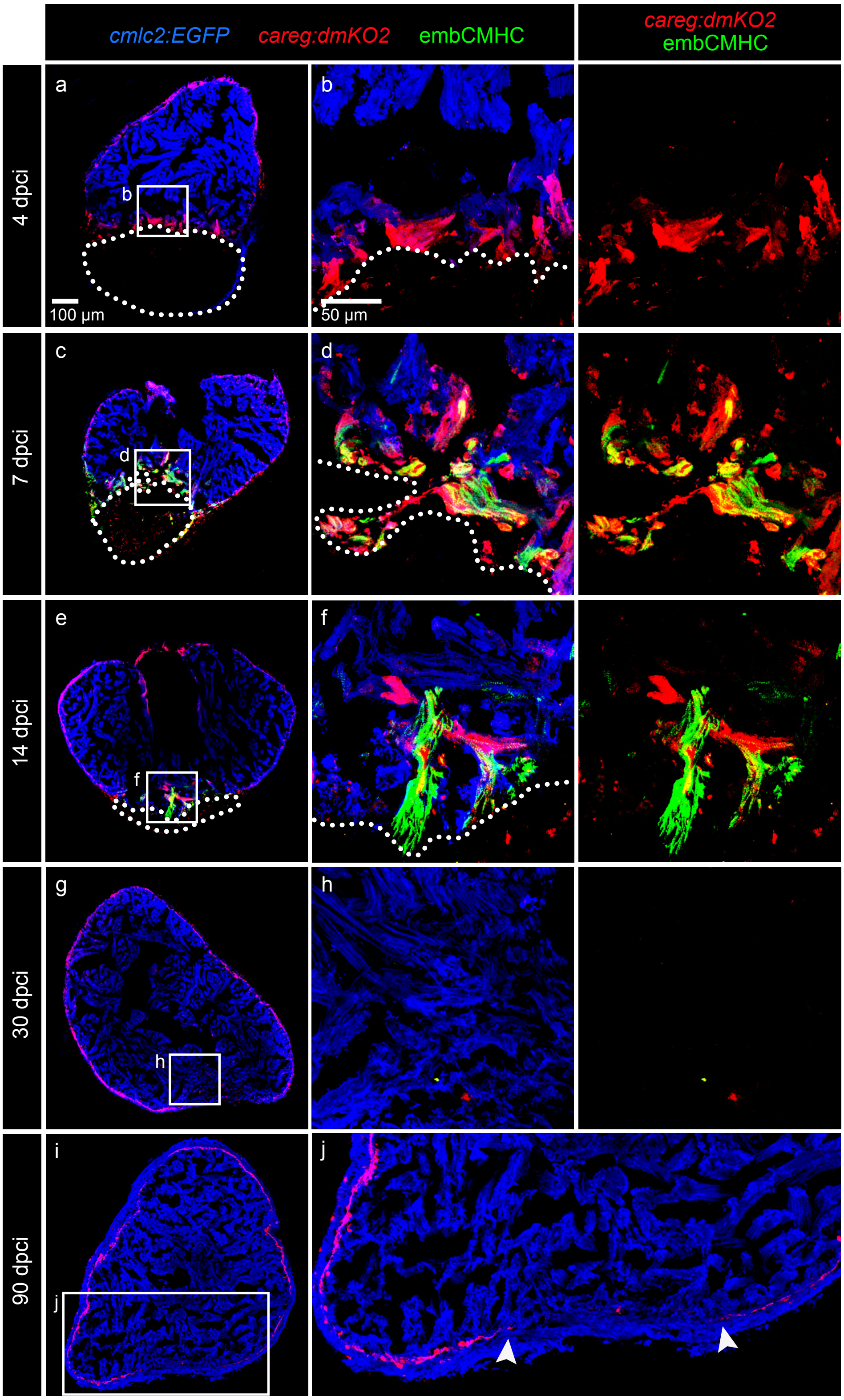

cmlc2:EGFP careg:dmKO2 embCMHC
careg:dmKO2
embCMHC
4 dpci
7 dpci
14 dpci
30 dpci
90 dpci
a
b
100 µm
50 µm
c
d
e
f
g
h
i
j

Supplementary Figure 3. *careg:dmKO2* is transiently expressed in the peri-injured myocardium during heart regeneration.

(a-j) Immunofluorescence staining of *careg:dmKO2;cmlc2:EGFP* heart sections labelled with antibodies against GFP (cardiac cells, blue) and embCMHC (green) at different time points after cryoinjury. N=4. (a-b) At 4 dpci, *careg:dmKO2*+ CMs emerge along the wound margin. (c-f) At 7 and 14 dpci, a large proportion of *careg:dmKO2*+ CMs activates the expression of embCMHC. (g-h) At 30 dpci, *careg:dmKO2* expression declines in the trabecular myocardium, but the primordial layer was not fully restored in the regenerated part of the ventricle. (i-j) At 90 dpci, the myocardium is completely regenerated, but the expression of *careg:dmKO2* is not completely reestablished in the subcortical region. The arrowheads indicate the position of the gap in the subcorical layer.

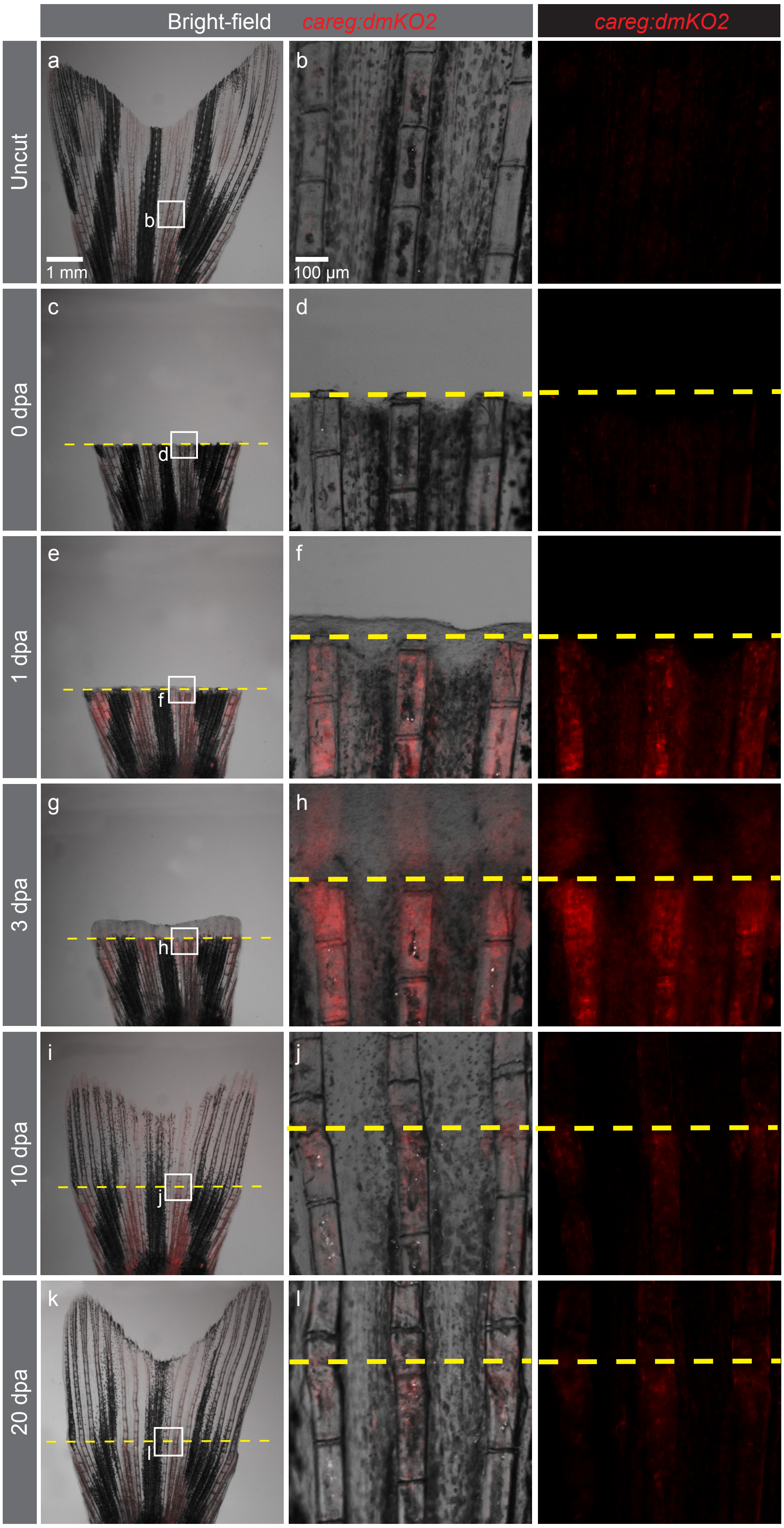

Supplementary Figure 4. *careg:dmKO2* is transiently expressed in the peri-injured stump during fin regeneration.

(a-l) Live-imaging of *careg:dmKO2* fins at different days post-amputation (dpa). Higher magnifications of framed areas show the region of the ampulation plane (yellow dashed line). N=4.

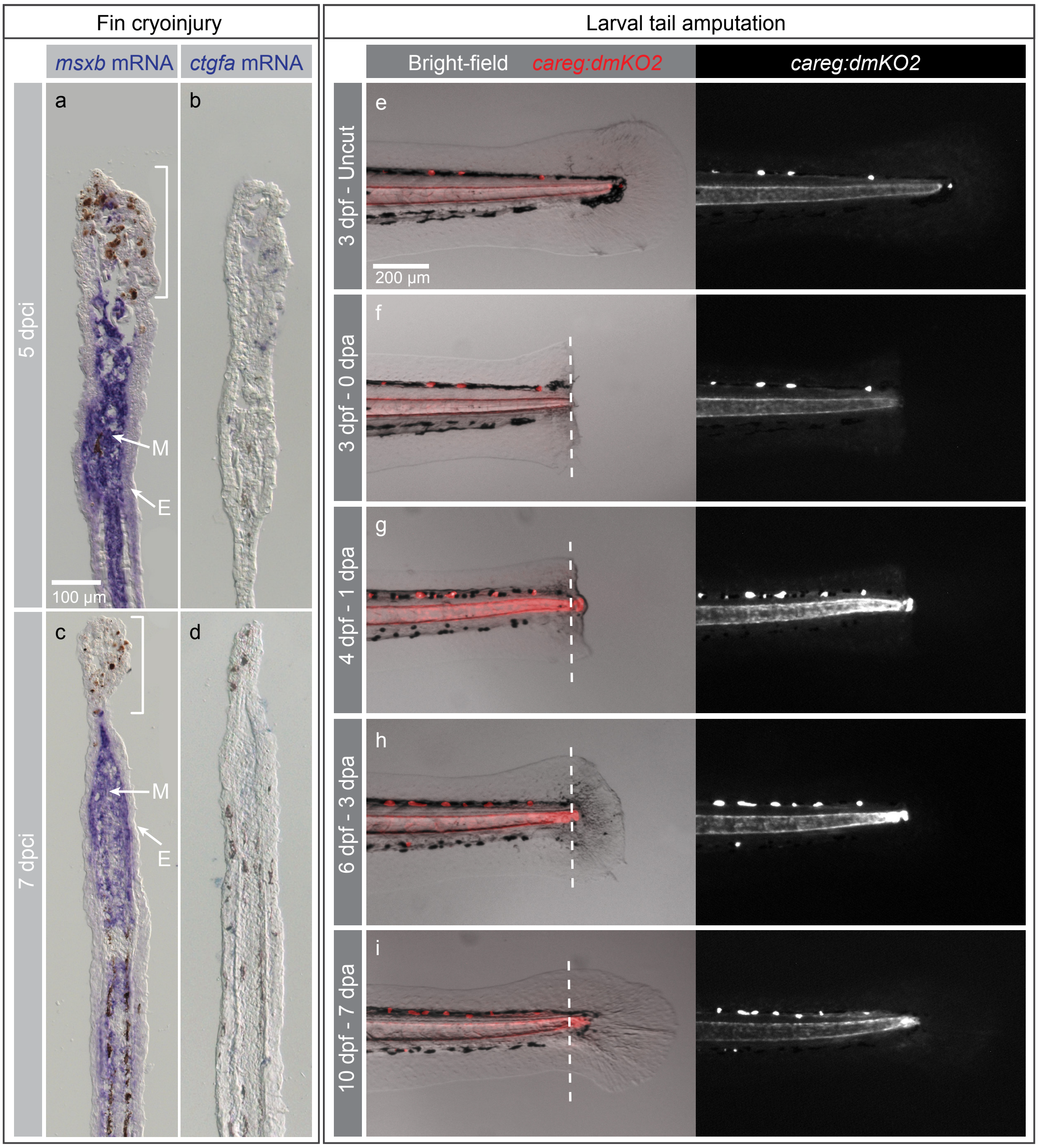

Supplementary Figure 5. The *careg* reporter is activated in other injury models of tissue regeneration in zebrafish.

(a-d) In-situ hybridization (purple) on longitudinal fin sections reveals that, as in the amputation model, the endogenous *ctgfa* gene is not upregulated in the regenerating fin after cryoinjury. (a, c) A probe against a blastema marker, *msxb*, was used as a positive control to detect the activated mesenchyme. (b, d) A *ctgfa* probe does not label the regenerating fin tissues. M, mesenchyme; E, epidermis. Brackets indicate the damaged tissue remaining at the tip of the cryoinjured stump. N=3.

(e-i) Live-imaging of the larval tail of *careg:dmKO2* fish at different days post-fertilization (dpf) and post-amputation. The transgenic reporter is constitutively expressed in the notochord. After amputation (dashed line), the expression of *careg:dmKO2* is markedly upregulated at the tip of the regenerating tail. N=6.

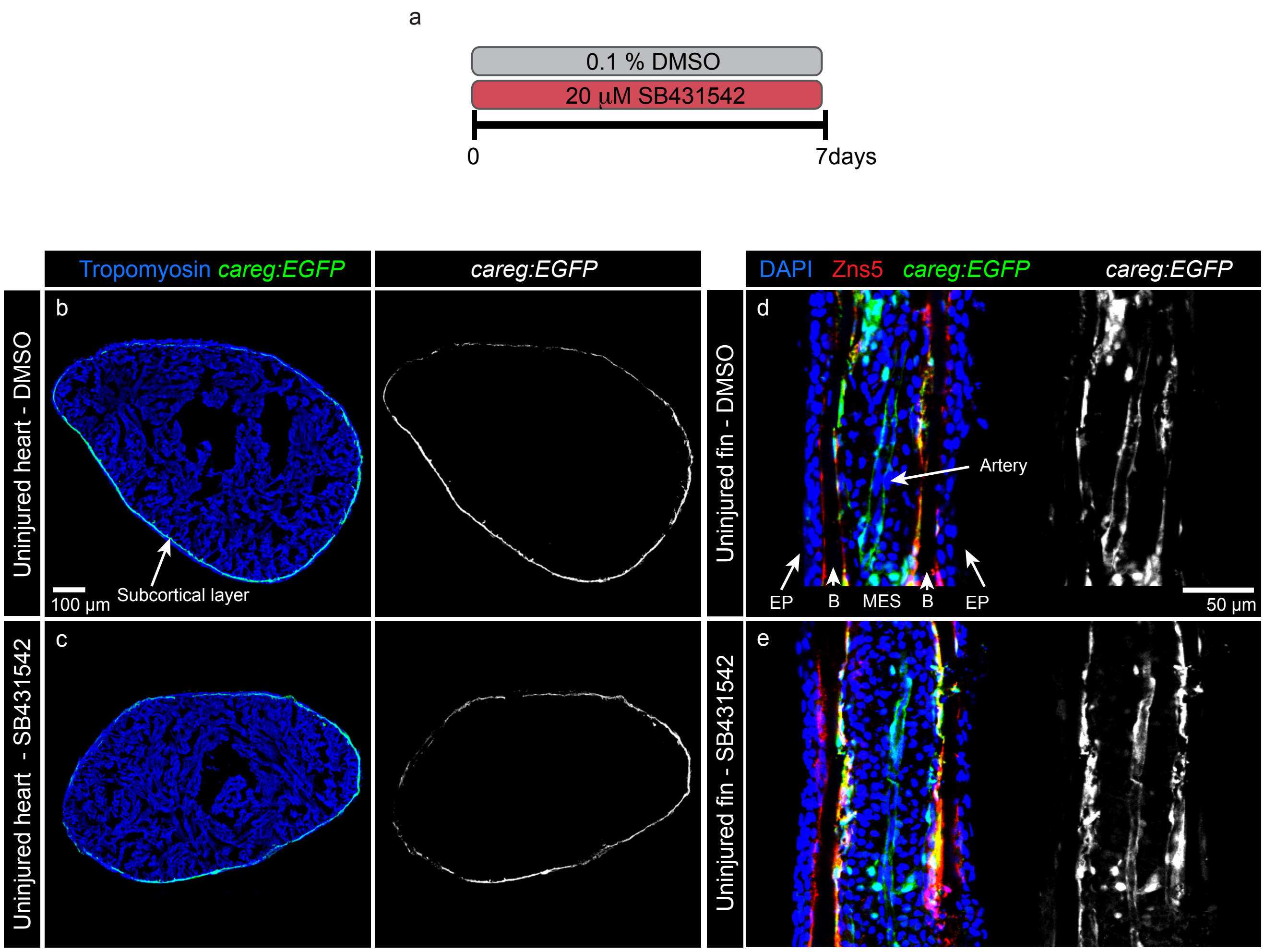

Supplementary Figure 6. Treatment of uninjured organs with the inhibitor of TGFβ/Activin-β signalling does not affect the homeostatic expression of *careg:EGFP*.

(a) Experimental design. Uninjured fish were treated with the inhibitor of TGFβ/Activin-β (20 µM SB431542) for 7 days. (b-c) Immunofluorescence staining of uninjured ventricles after 7 days of SB431542 treatment. The expression of *careg:EGFP* in the subcortical layer is similar in control and treated fish. (d-e) Longitudinal sections of rays in uninjured fins. Osteoblasts are marked with Zns5 antibody (red). They cover the surface of acellular bone matrix. In both control and treated specimens, *careg:EGFP* is expressed in intraray osteoblasts, in a few scattered MES and the artery. EP, epidermis; MES, mesenchyme; B, accelular bones. N=4.

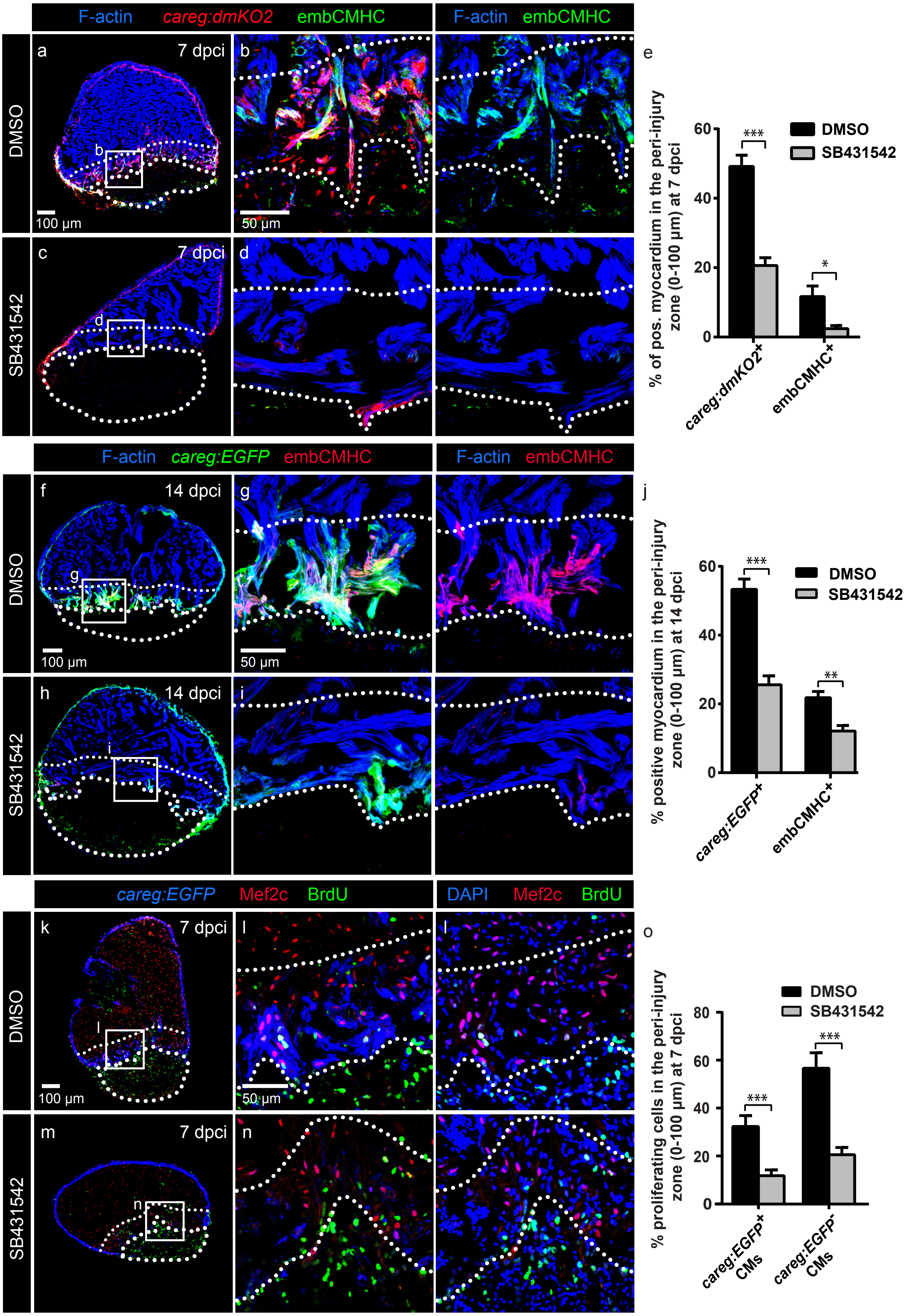
F-actin careg:dmKO2 embCMHC
F-actin embCMHC
DMSO
SB431542
a
7 dpci
b
100 µm
50 µm
c
7 dpci
d
e
% of pos. myocardium in the peri-injury zone (0-100 µm) at 7 dpci
DMSO
SB431542
***
*
careg:dmKO2+
embCMHC+
F-actin careg:EGFP embCMHC
F-actin embCMHC
f
14 dpci
g
h
14 dpci
i
j
% positive myocardium in the peri-injury zone (0-100 µm) at 14 dpci
**
careg:EGFP+
careg:EGFP Mef2c BrdU
DAPI Mef2c BrdU
k
7 dpci
l
m
n
o
% proliferating cells in the peri-injury zone (0-100 µm) at 7 dpci
careg:EGFP+ CMs
careg:EGFP- CMs

Supplementary Figure 7. The inhibition of TGFβ/Activin-β signaling suppresses CM dedifferentiation and proliferation at the injury-abutting zone during heart regeneration.

(a-d, f-i) Immunofluorescence staining of ventricles at 7 dpci (a-d) or 14 dpci (f-i) treated with DMSO or 20 µM SB431542 labelled with antibodies against fluorescent proteins and embCMHC. The intact myocardium is detected by F-actin staining (blue). The upper dotted lines indicate the 100 µm-thick margin of the myocardium along the injury border.

(e, j) Percentage of positive myocardium for the *careg* reporter or embCMHC in a 100 µm-wide margin of the regenerating myocardium in control or SB431542-treated hearts. $N \geq 5$. ***$p < 0.0001$, **$p < 0.01$, *$p < 0.01$; unpaired t-test. Error bars correspond to standard error of the mean (SEM).

(k-n) Immunofluorescence staining of *careg:EGFP* ventricle at 7 dpci treated with DMSO or 20 µM SB431542 labelled with antibodies against GFP (blue), Mef2c (cardiac nuclei, red) and BrdU (green).

(o) Percentage of BrdU+ CMs in a 100 µm-wide margin of the regenerating myocardium in control or SB431542-treated hearts. $N \geq 5$. ***$p < 0.0001$; unpaired t-test. Error bars correspond to standard error of the mean (SEM).

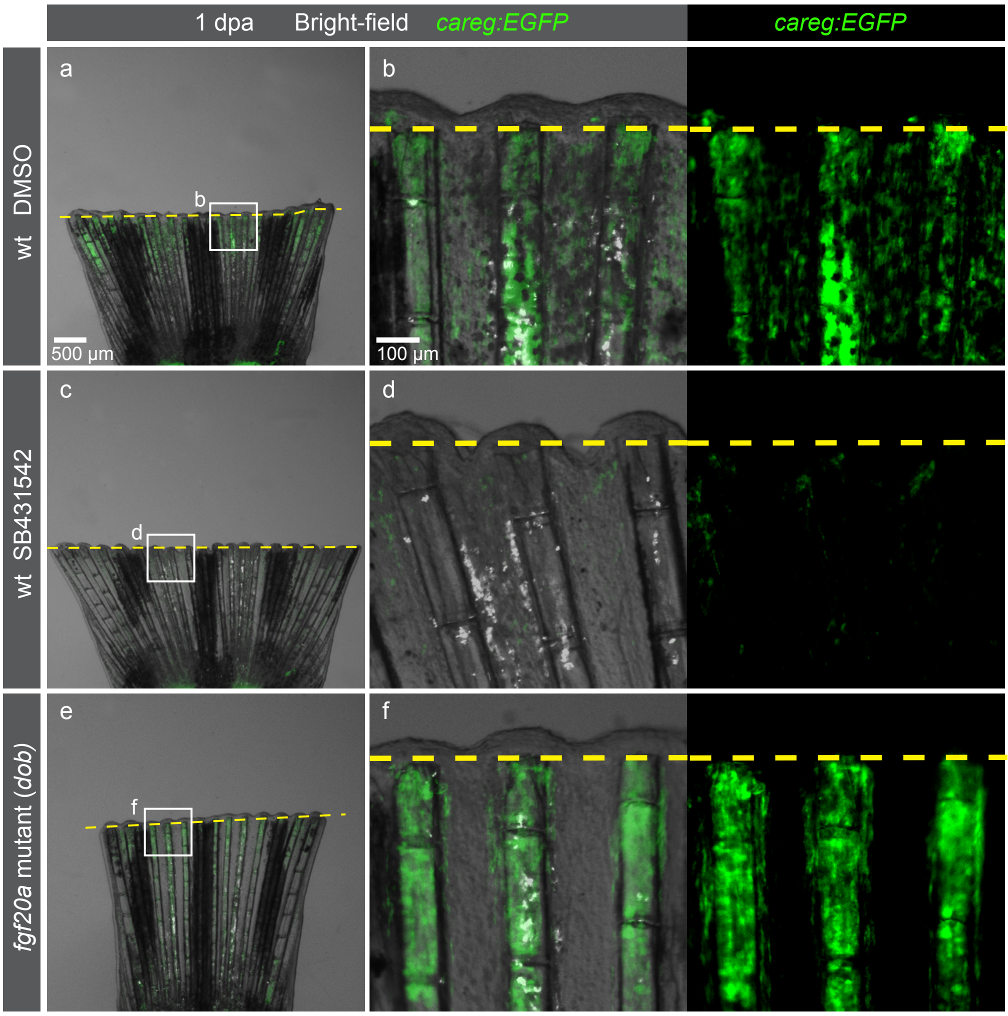

Supplementary Figure 8. Despite an absence of the regenerative outgrowth, *dob* mutant fins normally induce *careg:EGFP* in the amputation-activated stump.

(a-f) Live-imaging of *careg:EGFP* fins at 1 dpa in a wild-type (wt) background treated with DMSO or SB431542, and in the *fgf20a* (*dob*) mutant background. *careg:EGFP* expression is suppressed in the stump by the inhibition of TGFβ/Activin-β signaling, but it remains normal in *fgf20a* mutant fins. N=4.

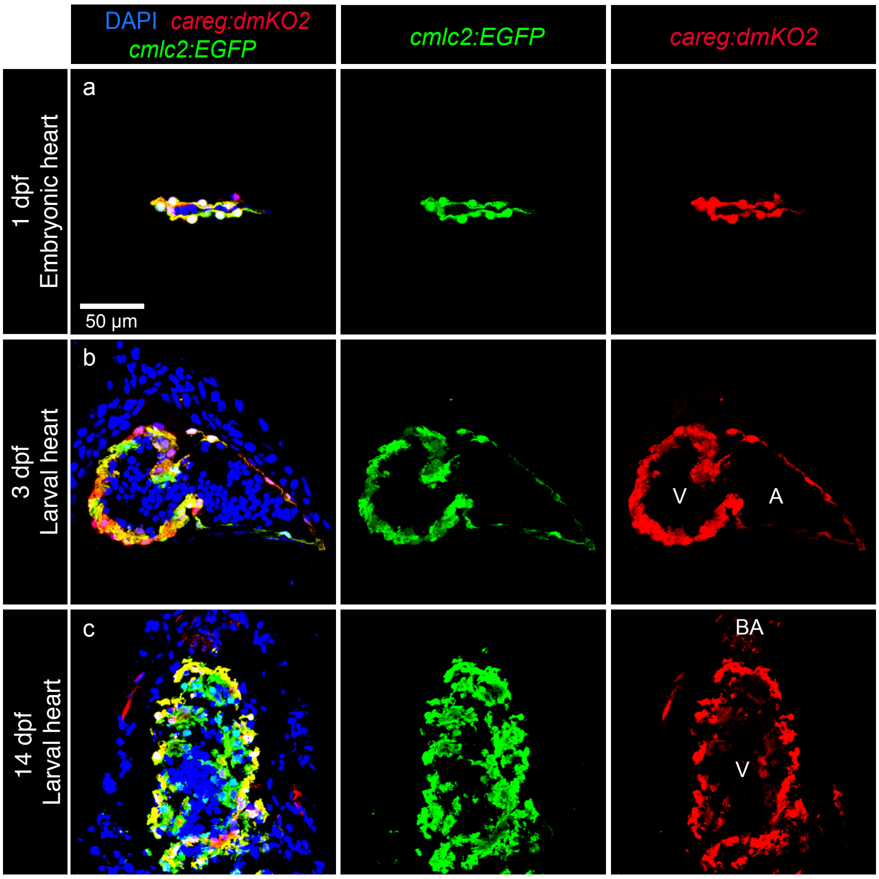

Supplementary Figure 9. Developmental dynamics of *careg:dmKO2* expression in the zebrafish heart.

(a-c) Longitudinal sections of *careg:dmKO2;cmlc2:EGFP* transgenic hearts at different time points during development, labelled with DAPI (nuclei, blue) and antibodies against GFP (green, cardiac cells) and KO2 (red). (a, b) *careg:dmKO2* is expressed in all embryonic cardiomyocytes at 1 and 3 dpf. (c) At 14 dpf, *careg:dmKO2* is maintained in the outer compact layer of the ventricle, whereas it is downregulated in the trabecular myocardium. *careg:dmKO2* is also expressed in the bulbus arteriosus. N ≥ 5. dpf, days post-fertilization; V, ventricle; BA, bulbus arteriosus; A, atrium.

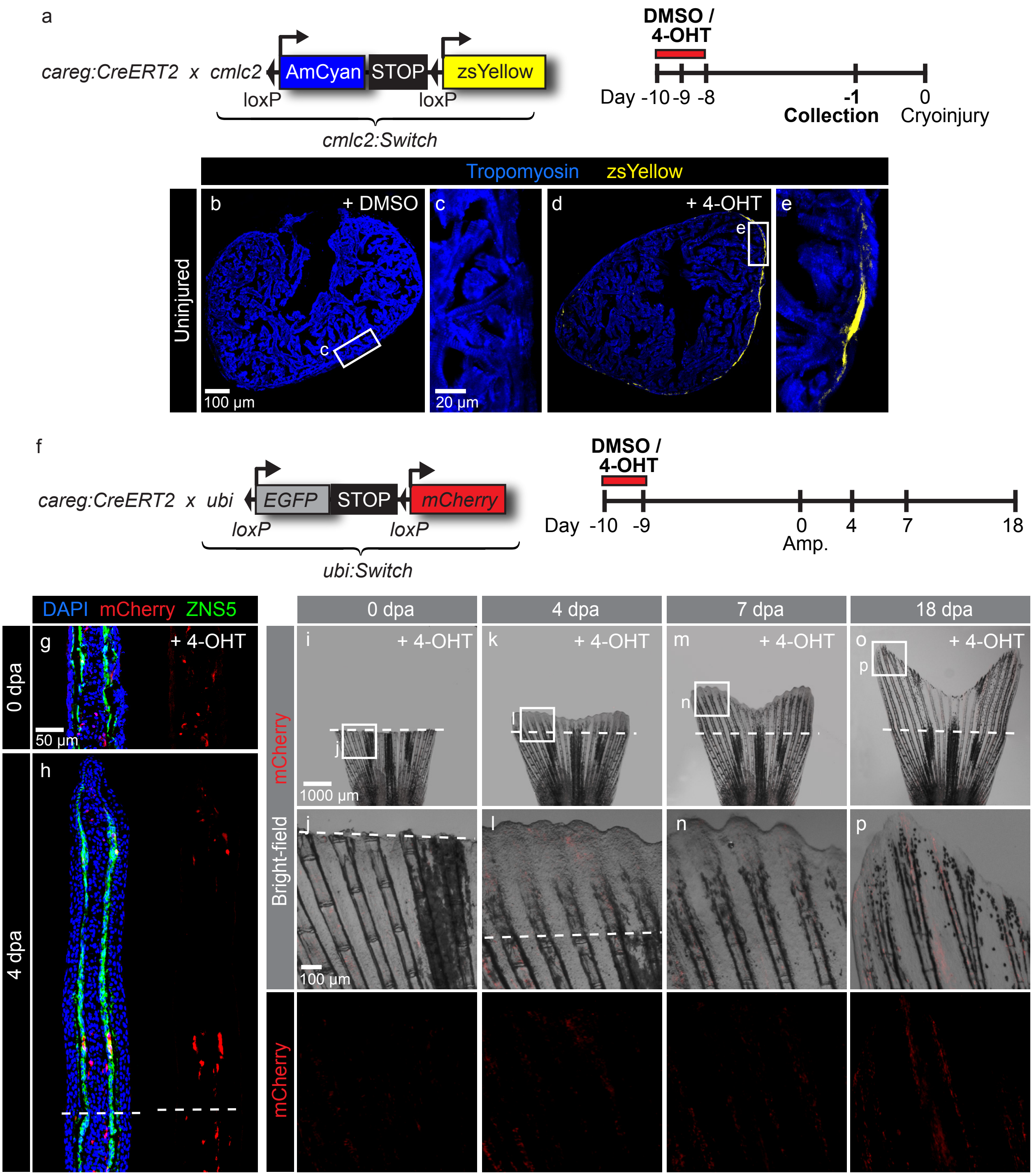

Supplementary Figure 10. Control experiments for *careg* lineage tracing analysis of heart and fin regeneration.

(a) Schematic representation of the transgenic strains and the experimental design for lineage tracing of *careg*-expressing primordial CMs during ventricle regeneration. (b-e) Immunostaining of uninjured ventricles treated with DMSO or 4-OHT using Tropomyosin (blue). Exposure to 4-OHT for 2 days is sufficient to label primordial CMs with zsYellow, as analysed at 7 days after the treatment (-1 day). (f) Schematic representation of the transgenic strains and the experimental design for lineage tracing of *careg*-expressing cells during fin regeneration. (g, h) Longitudinal fin sections at 0 and 4 dpa immunostaining for mCherry (red) and Zns5 (green). Exposure to 4-OHT for 1 day results in labelling of a few osteoblasts. (i-p) Live imaging of fins at different dpa after treatment with 4-OHT reveals only a few mCherry labelled cells in the stump and the regenerate. N $\geq$ 4.
